# Immersive cultural heritage digital documentation and information service for historical figure metaverse: a case of Zhu Xi, Song Dynasty, China

**DOI:** 10.1186/s40494-022-00749-8

**Published:** 2022-09-23

**Authors:** Zhanling Fan, Chongcheng Chen, Hongyu Huang

**Affiliations:** 1 Key Laboratory of Spatial Data Mining and Information Sharing of MOE, Fuzhou University, Fuzhou, 350108 China; 2 College of Computer and Data Science, Fuzhou University, Fuzhou, 350108 China; 3 Academy of Digital China (Fujian), Fuzhou University, Fuzhou, 350108 China

**Keywords:** Zhu Xi, Metaverse, Cultural heritage, Digital documentation, Virtual reality, Tourism service

## Abstract

Cultural heritage is closely linked with individual historical figures, who become a key focus for cultural tourism. Confucianism laid the foundation for much of Chinese civilization, and Confucius and Mencius have been studied extensively and have been influential in many parts of the world. Zhu Xi, the founder and master of Neo-Confucianism (an important part of the Mount Wuyi world cultural heritage), has received less research attention. With the big bang of the metaverse, research on the immersive cultural heritage digital documentation and information service for historical figures has become a new perspective. This paper presents a metaverse-based digital documentation framework for historical figures. This framework addresses the digitization of multimodal data for cultural heritage and adapts to the needs of metaverse presentations. For cultural research and cultural preservation, the paper has explored a combined contactless virtual and real cultural heritage experience system. For tourists, we intend to develop an immersive and holistic cultural tourism information service before, during and after the tour. In particular, we have also developed the Zhu Xi metaverse system. This is a step forward in the construction of a metaverse of historical figures.

## Introduction

Traditional culture is the soul of a nation, and as the creators of that culture, historical figures are important for its continuation. Rich cultural connotations, vivid historical records, and a wide variety of existing cultural heritage are precious resources for cultural tourism. Confucianism is a significant part of Chinese civilization, and Confucius and Mencius have been explored extensively. Zhu Xi, the founder and master of Neo-Confucianism, which is the core cultural element of the Mount Wuyi (List No. 911) world cultural heritage site, has received scant research attention. This doctrine played a dominant role in Eastern and South-Eastern Asian countries for many centuries and influenced philosophy and government over much of the world [1].

With the growing boom in tourism, the conflict between tourism development and cultural heritage preservation has become increasingly acute. New technologies—including three-dimensional (3D) laser scanning, unmanned aerial vehicle (UAV) photography, panoramic picture, panoramic video, extended reality (XR), and other digital museum technologies—new tools are being widely used in the digitization of cultural heritage. For example, 3D laser scanning technology (one of the powerful technologies for measuring and recording structures and landscapes [2, 3]) has been applied to the Hakka Tulou [4], Baroque masonry churches [5], nuraghe in Sardinia [6], and the Shukhov hyperboloid tower [7]. A UAV system can generate digital orthophoto maps (DOMs), digital surface models (DSMs), digital terrain models (DTMs), and 3D models [8]. It is faster than airborne and spaceborne remote sensing [9, 10]. Panoramic picture or video technology can provide an immersive expression of cultural heritage based on real picture or video capture, and intangible cultural heritage elements can be integrated into the virtual display [11]. With the advent of the experience economy, virtual reality (VR) has seen continuous updates and breakthroughs [12]. Research has shown that during the period of COVID-19, panoramic virtual tourism provided a decompression effect for people unable to travel [13]. The metaverse (a portmanteau of “meta” and “universe”) is a hypothesized iteration of the internet, supporting persistent online 3D virtual environments through conventional personal computing [14, 15], as well as virtual and augmented reality (AR) headsets [16]. The development of metaverse must go through three sequential stages, namely (I) digital twins, (II) digital natives, and eventually (III) co-existence of physical-virtual reality [17].

For historical figures, the aforementioned technologies offer new perspectives for their digital recording, preservation and presentation. Prior to this research, Zhu Xi-themed cultural resources had not been fully digitized and stored. Thanks to the attention of the local government, the restoration and tourism development of Zhu Xi’s cultural heritage will enter a high-speed stage. Documentation of the original features is essential before irreparable damage is done. Methods of acquiring cultural heritage with high-fidelity and high-precision are priority tasks. In addition, the integration and unified use of digitized cultural heritage information from multiple sources is a topic worthy of study. In terms of digital presentation, combining the information technology with the content of cultural heritage to better tell its story and personalize its presentation for different audiences is another great challenge. This paper will explore the above issues.

## Materials and methods

### Research aim

Methodological tasks geared toward high-fidelity and high-precision acquisition of cultural heritage. This study explores a digital documentation framework on the cultural heritage of historical figures. It proposes to introduce immersive digitization techniques to address the digitization of multimodal resources. Based on digital documentation of cultural heritage, this paper designs an information service platform for the cultural heritage of a historical figure. It also incorporates parts of the metaverse to provide personalized services for different audience groups. This is our exploration to solve the contradictory problem of cultural heritage preservation and development.

### An overview of Zhu Xi and materials

Zhu Xi (1130–1200)—also known by his courtesy name Yuanhui (or Zhonghui) and self-titled Huiweng—was born in Youxi County, Fujian Province. He was a famous calligrapher, historian, philosopher, politician, and writer in the Southern Song Dynasty, a representative of Min scholars, and the most outstanding master of Confucianism since Confucius and Mencius. During his life, he served as a government official for multiple times. Most of his adult life was spent lecturing and writing. In his later years, he settled in Kaotting and was buried in the Huangkeng, Jianyang. Later generations respectfully called him Master Zhu, Zhu Zi, or Zhu Wengong. His Neo-Confucianism became the official philosophy of the Yuan, Ming, and Qing Dynasties, which still has a wide and profound influence.

**Fig. 1:**
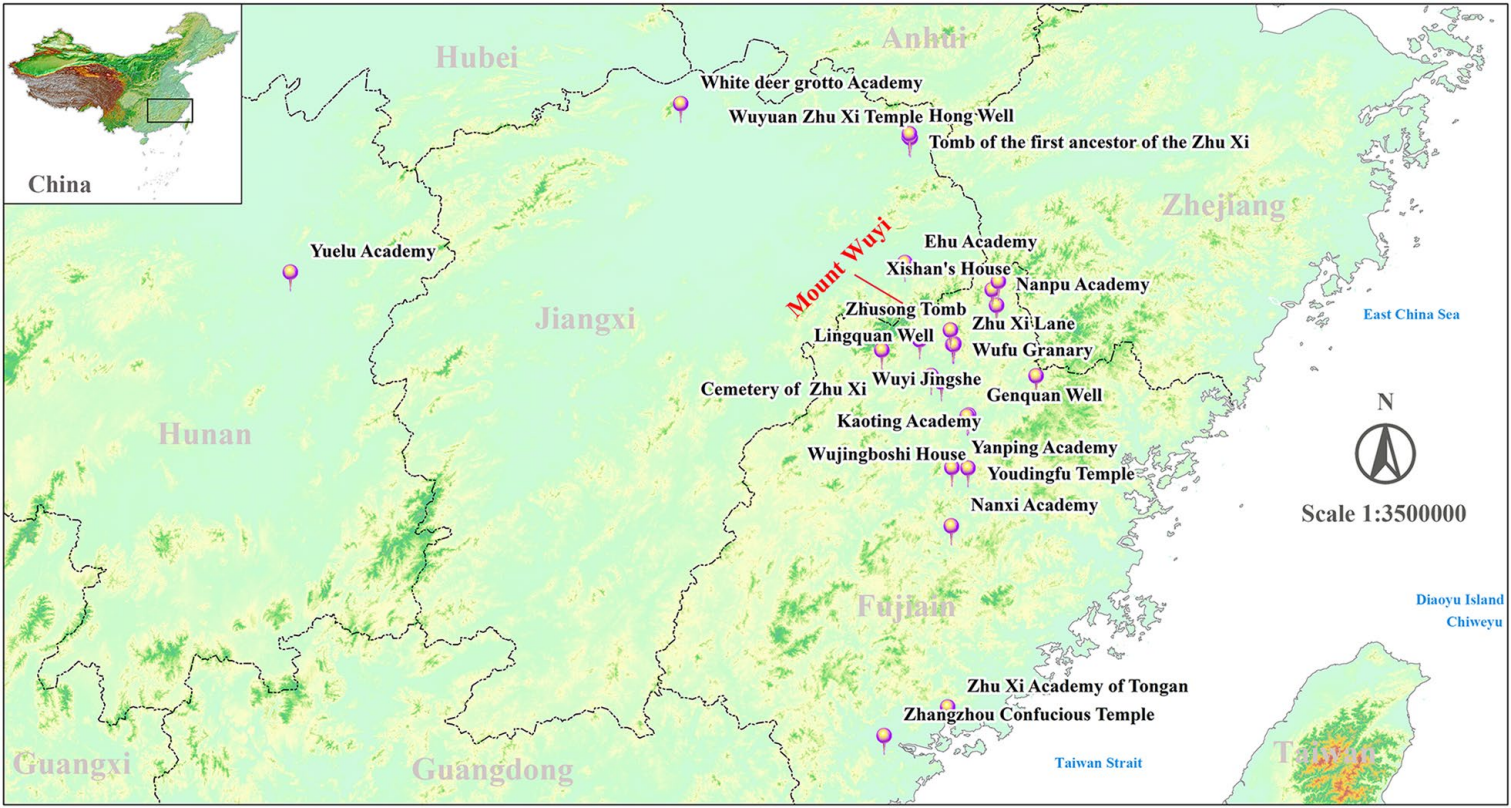
Distribution map of major cultural heritage sites related to Zhu Xi in Southern China

The cultural heritage related to Zhu Xi is very rich. For example, in Nanping, Fujian Province, there are 135 tangible cultural heritages related to Zhu Xi, including two major historical and cultural sites protected at the national level, 11 sites protected at the provincial level, and 30 sites protected at the county level. Zhu Xi’s footprints cover Sanming City, Xiamen City, and Zhangzhou City of Fujian Province, as well as some cities in the neighboring Jiangxi and Hunan Provinces (Shown in Fig. 1). Equal in importance to the tangible cultural heritage, the intangible heritage related to Zhu Xi includes Zhu Xi Festival, wedding ceremony, teacher worship ceremony, capping ceremony (Four Rites), and the Longyu opera, which have been listed in the provincial level intangible cultural heritage of Fujian Province. The following materials are used in this study.

- Research literature: examples profiles, biographies, genealogies, historical titles, historical pictures, anecdotes, theoretical research, teachings, publications, calligraphy, etc. Much of this material exists in fragmentary form in text, pictures, etc. And access to them is time-consuming.
- Tangible cultural heritage: examples Kaoting Academy, White deer grotto Academy, etc. The conservation status of cultural heritage is not uniform in different locations. Some that are incorporated into the management of tourist attractions are better protected, such as Wuyi Jingshe and White deer grotto Academy. Some are in an unprotected state.
- Intangible cultural heritage: examples Zhu Xi Festival, wedding ceremony, teacher worship ceremony, capping ceremony, etc.
- Tourist elements: examples attractions, itineraries, travelogues, points of interest (e.g., hotels, restaurants, service facilities), creative products, etc. These are closely related to cultural heritage tourism.

### Digital documentation methods for historical figures

**Fig. 2:**
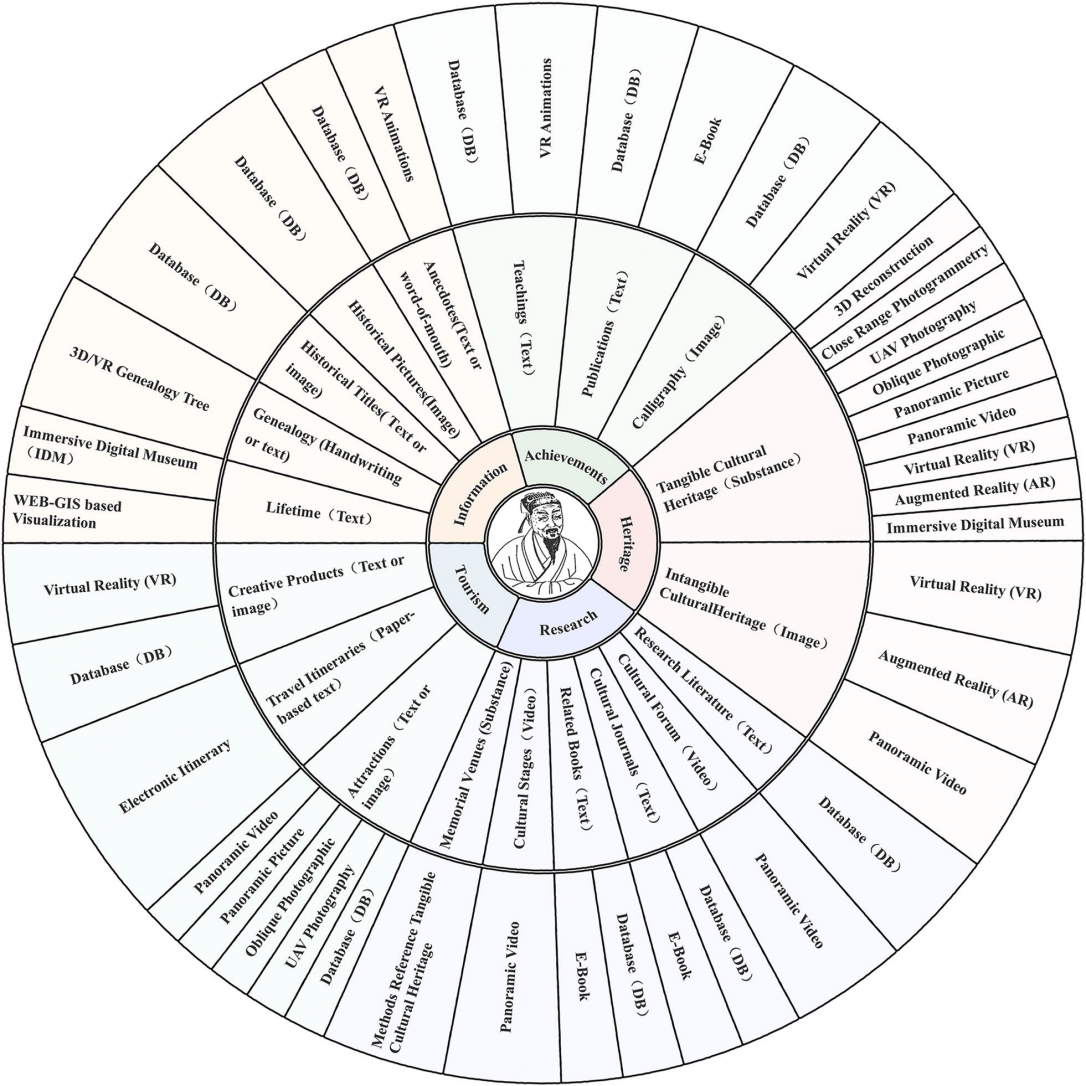
Digital documentation framework for historical figures

Via extensive data collection and collation, we present a metaverse-based digital documentation framework for historical figures (MDF-HF) (Shown in Fig. 2), which comprises the following five areas.

**Fig. 3:**
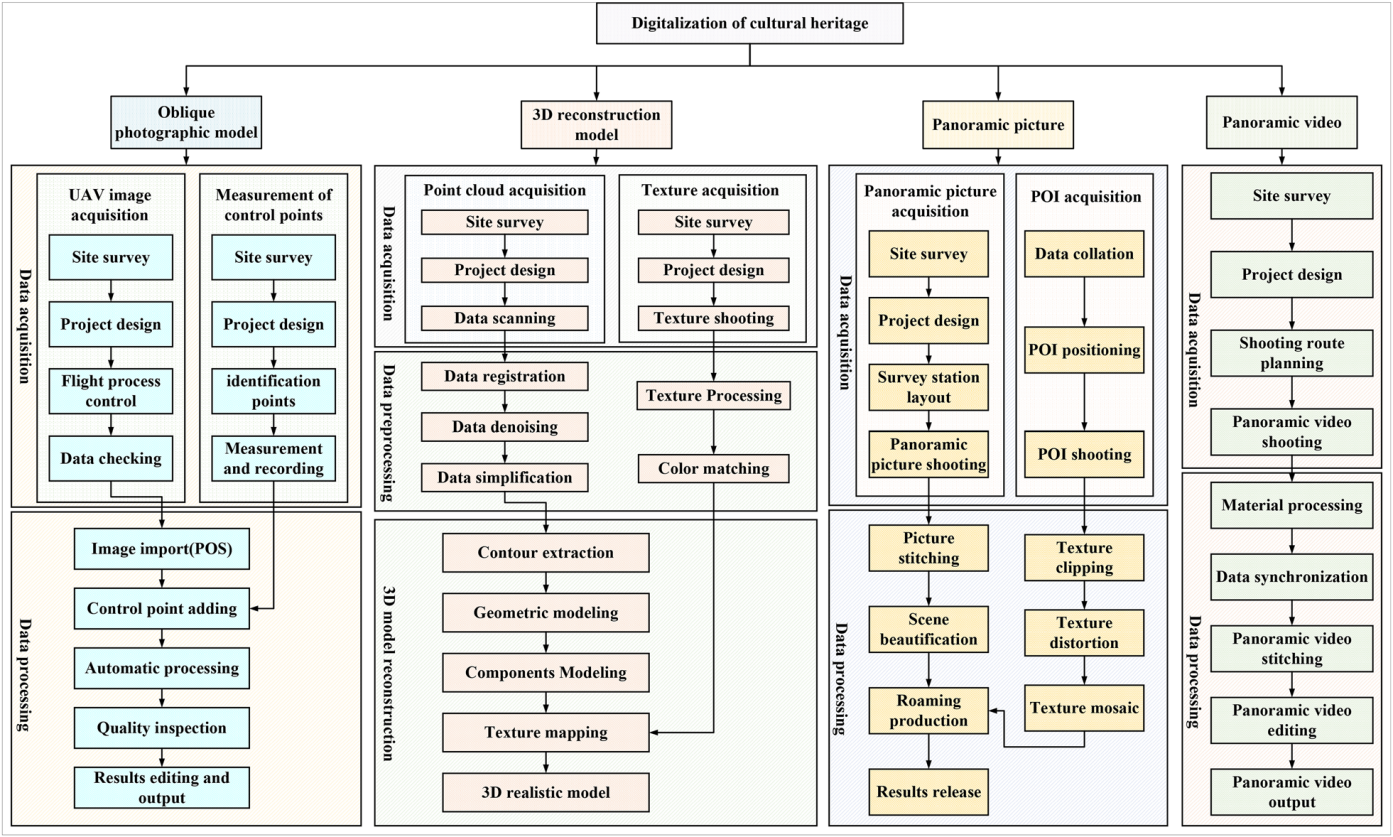
The processing flows of the main digitization methods of cultural heritage

- Basic information. The contents include profiles, biographies, genealogies, historical titles, historical pictures and anecdotes. Mostly, we organize and digitize the information in depth and store it in a database. Given that lifetime information has a temporal and spatial character, Web GIS (geographic information system) based on visualization and immersive digital museum (IDM) technology is employed to present it. 3D/VR genealogy trees are more suitable for visualizing the genealogy information. To enhance digital natives’ contents, we also employ VR animation technology to recreate the anecdotes of historical figures.
- Figure achievements. Historical figures had accomplishments in different fields. Taking Zhu Xi as an example, these achievements are theoretical research, teachings, publications, and calligraphy that have survived. Recorded in handwritten (or textual) form, these theories can be abstract, obscure, and difficult to grasp. To make its essence more accessible to a wider audience, we employ technologies such as VR, VR animation and E-Book to digitize it.
- Cultural heritage. Historical figures have left behind a substantial cultural heritage, both tangible and intangible. These items are the basis of the digital twins. For tangible cultural heritage, we propose a digital approach system that integrates reality and virtualization. The realistic approach includes close range photogrammetry, UAV photography, oblique photographic, panoramic picture or video. Virtual methods comprise 3D reconstruction, VR, AR, immersive digital museum. For intangible cultural heritage, we use VR, AR and panoramic video methods for representation.
- Posthumous research. The significant influence of historical figures has inspired researchers to establish professional research institutions, organize various forums, accumulate a wealth of research results, develop a vast number of promotional products (such as stages), build a mass of memorial venues and publish a large amount of cultural information. Cultural stages and cultural forums are recorded through panoramic video technology, cultural journals are produced as E-Books, and the digitization of memorial venues can draw on the system of digital tangible cultural heritage.
- Tourism elements. Historical figures play an irreplaceable role in tourism as important tourist IP (intellectual property) in cities, and likewise, the development of tourism will further facilitate the research and promote the culture of historical figures. For tourist attractions, we use UAV photography, oblique photographic, panoramic picture or video technology instead of the traditional text and picture presentation, and propose personalized electronic itineraries. Moreover, some creative products are digitized in VR. The processing flows of major methods is shown in Fig. 3.

#### Oblique photographic model

The real environmental data surrounding cultural heritage plays an important role in the visualization of that heritage. Through a mosaic of an oblique photography model and a 3D reconstruction model, the overall experience of the cultural heritage—from global to local—can be presented. Oblique photography is used to capture the environment surrounding the cultural heritage, and the activities involved in this data acquisition and processing are described below.

The purpose of controlled survey is to determine the absolute position of an object in geographic space. The ground control points were set to intervals between 400 and 800 m according to the geographic environment. The field control points were marked with red-white crossed polyvinyl chloride (PVC) panels (40 cm × 40 cm). Absolute geographic location accuracy less than 1 m and relative accuracy less than 10 cm were achieved. Aerial photography time was limited to 2–3 h around noon—that is, 11:00–13:00 (Beijing time). The wind was limited to less than level 3. The ground resolution was set between 0.02 and 0.05 m. The special objects in the key areas had a high resolution of 0.02 m and a relative flight height of 120 m, with a resolution of 0.05 m for some hilly areas with large fluctuations and a relative flight height of 300 m. Directional overlap was set to 80% while side overlap was set to70%. The UAV platform used in the project was the DJI MATRICE 600 PRO, and the image acquisition camera was a SHARE 101S. After a single aerial campaign was completed, the coarse collage of aerial photographs was checked using the Pix4D software rapid detection module, and low-precision results were generated to ensure that the aerial photography task completed all of the work designed in the shooting scheme. The photogrammetric software Pix4DMapper was used for data processing. The main steps included image and POS (position and orientation system) control data import, control point import, automated aerial triangulation calculation, quality data inspection, result editing and output.

#### 3D reconstruction model

The 3D model reconstruction was the most important work of this project, the main data source for digital documents and the visual virtual expression of the cultural heritage. We completed the 3D scanning and reconstruction of realistic models of the Zhu Xi cultural heritage sites listed in Table 1.

**Table 1:** List of major cultural heritage of Zhu Xi

County	Name
Yanping,Nanping City,Fujian	Yanping Academy,Youdingfu Temple
Jianyang,Nanping City,Fujian	Kaoting Academy,Cemetery of Zhu Xi,Tomb of Zhu Xi's mother
Wuyishan,Nanping City,Fujian	Xingxian Academy,Wuyi Jingshe,Zirang Building, Zhu Xi Lane,Wufu Granary,Lingquan Well, Camphor tree planted with Zhu Xi,Zhusong Tomb
Jianou,Nanping City,Fujian	Wujingboshi House,Jianou Confucious Temple, Genquan Well
Pucheng,Nanping City,Fujian	Nanpu Academy,Xishan's House,Zan Tiren's house
Zhenghe,Nanping City,Fujian	Yungen Academy
Youxi,Sanming City,Fujian	Nanxi Academy
Tong'an,Xiamen City,Fujian	Zhu Xi Academy of Tongan
Xiangcheng,Zhangzhou City,Fujian	Zhangzhou Confucious Temple
Wuyuan,Shangrao City,Jiangxi	Hong Well,Tomb of the first ancestor of Zhu Xi. Wuyuan Zhu Xi Temple
Qianshan,Shangrao City,Jiangxi	Ehu Academy
Lushan City,Jiujiang City,Jiangxi	White deer grotto Academy
Yuelu,Changsha City,Hunan	Yuelu Academy

Data acquisition included point clouds and textures. We used a 3D laser scanner (RIEGL VZ-400) to obtain a 3D point cloud. The optimal scanning distance was 25 m, with a point cloud interval of 10 mm. The device automatically scanned and stored data before being moved to the next station. Each site was scanned from multiple positions to ensure full coverage. The texture was critical to establishing a realistic 3D model. Textures were collected using a digital SLR camera (Nikon D800 and Nikon 24–105-mm f/4) and included features such as building facades, windows, doors, corridors, and columns. Point cloud data preprocessing included noise removal, data registration and data simplification. Texture data were processed by clipping, warping, distortion, splicing and color matching. Model reconstruction including contour model and component models. Take building model as an example, the wall model was first obtained by stretching, offsetting, and modifying the 2D drawing. Next, the 3D model of each roof was built according to the views of the roof. Based on CAD drawings, a 3D model of the overall model was obtained by stretching, chamfering, and other tools. The windows, doors, columns, and other smaller models were built based on component measurement data. Finally, the models were merged and the connection between the walls and the roof was modified accordingly. A realistic 3D model was constructed using texture mapping on the texture-less model.

**Table 2:** Main parameters setting of panoramic camera

Capture parameters	Detailed description
shooting mode	Manual mode (M)
aperture setting	F8-11
ISO speed	100–400
focus setting	(Infinity)
overlap rate	30–50%
white balance	Automatic white balance
resolution ratio	No less than 20 million pixels

#### Panoramic picture

Traditional pictures and videos can only provide local scenes with a limited field of view. To integrate virtual and real expression of cultural heritage, we used panoramic pictures and videos to show the whole real scene. After collecting panoramic pictures and local points-of-interest information, an interactive 3D space scene was simulated to offer the expression of real scene information from the cultural heritage site.

Data acquisition included collecting panoramic pictures and local points-of-interest. To obtain both panoramic and local scenes of the cultural heritage site, panoramic picture data were acquired both from the air and on the ground. The aerial panorama was taken by a UAV (DJI Mavic Pro), and the ground panorama was taken using a digital SLR camera (Nikon D800, Nikon AF16mm f/2.8D). The parameters setting of the panoramic camera was listed in Table 2. To keep the acquired image scene clean and tidy, clear weather and high visibility were necessary. It was also important to minimize the number of people present and to avoid peak visiting times to reduce interference from tourists and residents. For outdoor panoramic shooting, sunny and well-lit times were chosen to keep the scene fresh and bright. For indoor panoramic shooting, a large light contrast between outdoors and indoors would result in some corners of the room too dark; this can be avoided by choosing an appropriate shooting time or by pulling up the curtains. After systematic collection of the cultural heritage data, a list of key points-of-interest was formed. The texture of the key points was gathered under sunny weather, but direct sunlight was avoided. The best time for image capture was 10:00–15:00. It was not suitable to shoot for texture at dusk. Data processing included picture stitching, scene beautification, roaming production, and releasing the results. During the stitching process, the control points were static objects; selection of moving objects such as trees and clouds was avoided. It was necessary to ensure that there were no missing scenes and the transitions occurred naturally. Roaming production included adding hotspots between scenes, map-making with radar scanning effects, scene music and interpretation, text information, and other multimedia elements. To keep the scene clear, the resolution of the scene was greater than or equal to 8000*4000 pixels. The initial angle of the scene was adjusted before adding scene hotspots to keep the maximum field of view and to avoid blurred pictures during browsing. When adding map radar, the size of the map player was the same as the size of the map to avoid issues such as hotspot offset, failure, or partial display of the map. The finished panoramic roaming was published in a generic HTML5 format accessible from multiple platforms.

**Table 3:** Questionnaire results of ZXCHIS used for user studies

Questions	Agree (%)	Not sure (%)	Disagree (%)
The site helps you to further understanding the Zhu Xi culture	95	4	1
The virtual experience of panoramic pictures or videos is attractive to you	93	6	1
Virtual scenes incorporating oblique models and 3D models can give you a better understanding of cultural heritage	90	7	3
The platform has an enhancing effect on the preservation and promotion of the cultural heritage of Zhu Xi culture	98	1	1
The tourism information in site is useful to you	91	7	2

#### Panoramic video

Recording dynamic panoramic videos is also an important way of presenting tangible and natural cultural heritage. Panoramic pictures can only display static scenes, while panoramic video was used for the resource acquisition of dynamic scenes such as the Zhu Xi Festival.

Before shooting, it was necessary to communicate with the sponsor and conduct an on-site inspection of the event, which was essential to understand the process and core links of the whole activity. A panoramic shooting route based on the results of the field survey was then designed; this included both fixed camera locations (on the ground) and mobile camera locations (in the air or on the ground). Independent audio recording was also important during the shooting process. The panoramic video was shot with six cameras working at the same time, so it was imperative to turn on the camera with a remote control to ensure that the remote control could govern all cameras. The camera used in the project was the GoPro OMNI. Two pieces of software, Kolor Autopano Video Pro (AVP) and Kolor Autopano Giga (APG), were used to stitch the panoramic video together. AVP was used to realize the pre-splicing of the panoramic video, and then APG completed the fine splicing of the panoramic video. Although the GoPro cameras used a remote control to synchronize video recording, there were still frame-level errors that had to be desynchronized. AVP is much better at processing pictures than videos; so it was necessary to convert stitched video files into picture sequences. The length and synchronization of the video could result in a different number of frames per image sequence. Therefore, appropriate quantities were deleted as the end to ensure that each image sequence had the same number. To ensure the clarity of the panoramic video, the splicing resolution was better than 8 K. Finally, depending on the needs of the different platforms, the panoramic video was output to multi-resolution, multi-format panoramic video products.

#### VR animation

There are many classic stories about Zhu Xi, which have a long oral and literary tradition. VR technology can create an immersive experience that allows people to understand these vivid and interesting stories. We have developed 10 VR animation stories about Zhu Xi, each of which lasted for 2–3 min. Story scripting is the essence of this work. The scene design should set out the architectural decorations, artefacts and urban street scenes of the period according to the dynasty, culture and context in which Zhu Xi lived. Part of the scene can directly use the existing Zhu Xi cultural relics. Six lenses are used to render the scene during the shooting stage, with a single lens rendering resolution of no less than 8 K.

#### Digital museum

The Zhu Xi Digital Museum is a virtual exhibition hall that integrates Zhu Xi’s life footprint, Neo-Confucianism achievements, historical stories, and cultural heritage. Without restriction of physical space, it has the advantage of being always accessible with fast update speed and low update cost. The digital museum with VR support can also provide a highly immersive virtual experience and intelligent voice tour function. The production of the Zhu Xi Digital Museum included virtual venue design, 3D model building, and virtual roaming production. A total of 10 main content sections are included.

### Information service design for historical figures

#### Design basis

**Fig. 4:**
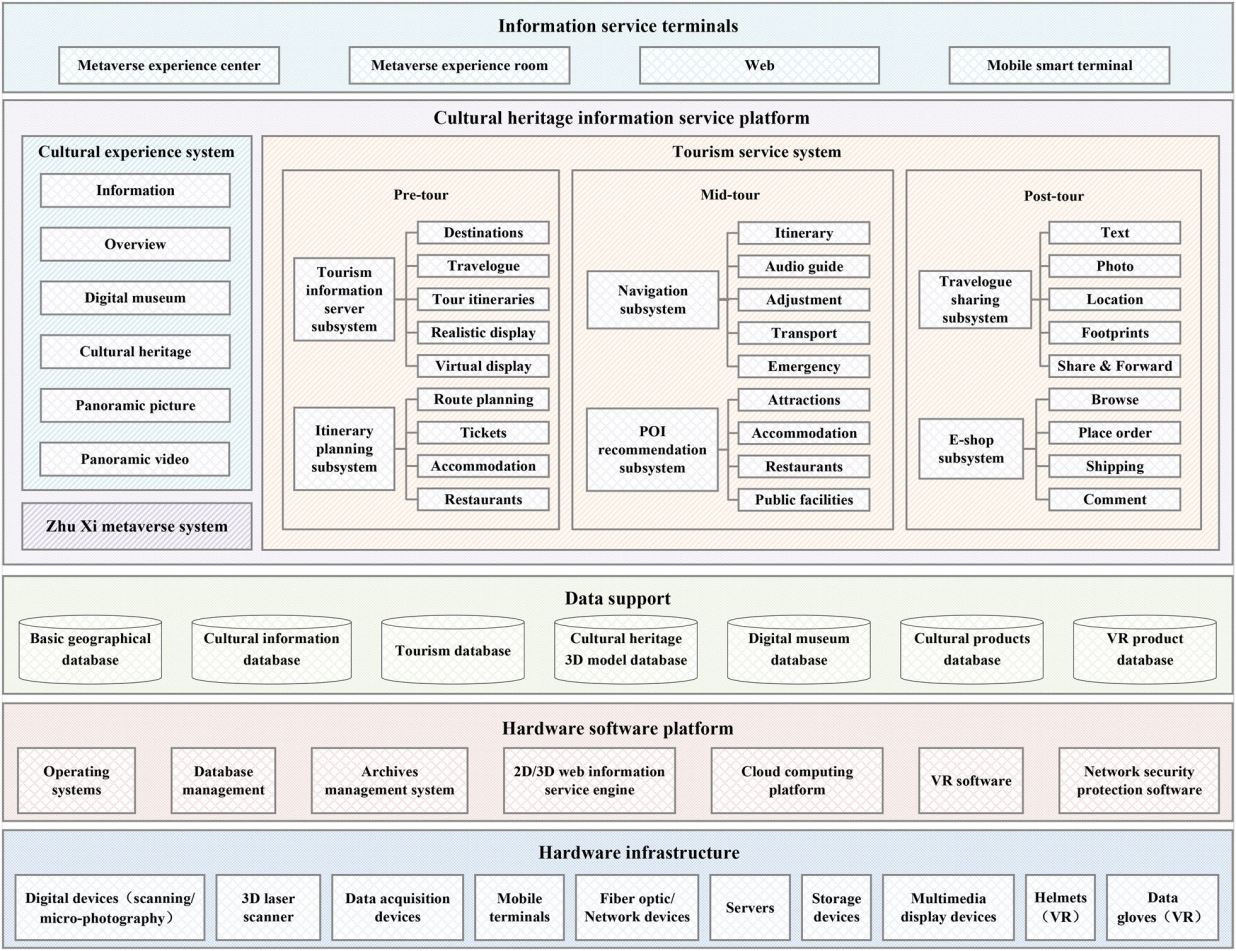
The structure of the cultural heritage information service platform

The first targeted audience of the cultural experience system is cultural researchers. Their attention is focused on result-based management and further cultural research. The presentation of research results and education is also a major part of their work. An online service that covers the entire travel process is a pain point, especially for self-drive tours. For school students and the public, the interaction of real and virtual environments through immersive metaverse technology can stimulate their interest in cultural experiences. The structure of the Zhu Xi cultural heritage information service platform (ZXCHIS) is illustrated in Fig. 4. The platform comprises systems of cultural experiences, tourist services and Zhu Xi metaverse. It is running on the internet and metaverse experience center. The details of the design are as follows:

#### Cultural experience system

The cultural experience system provides access, browsing and immersive experiences of resources on historical figures. Different modules are designed in the paper for different contents.

1. Information: Timely report on research progress is essential to facilitate cultural heritage communication. This module provides information such as news, official announcements, theoretical research, and cultural exchange of related activities. It achieves multi-platform publishing, reduces the workload of maintenance staff and supports forwarding to third-party platforms to expand the display channels of announcement contents.
2. Overview: This module displays culture related to Zhu Xi through text, picture, video, and animation, with content about Zhu Xi’s cultural connotation, chronology, Neo-Confucianism, classical works, stories, political concepts, educational thoughts, cultural heritage, publications, dissemination of Neo-Confucianism, four rites, and songs. Here you can find almost anything related to Zhu Xi.
3. Digital museum: The digital museum supports automatic roaming and autonomous navigation, with the effect of changing scenery along each step. Clicking on hotspots provides detailed description or multimedia information browsing, giving viewers a rich interactive experience. With the museum guide map, it supports fast switching between navigation scenes. An audio guide accompanies each scene. Combined with gyroscope-enabled 3D glasses, it provides an immersive viewing experience.
4. Cultural heritage: This module combines a 3D digital model, remote sensing images, topographic information, and oblique photographic models of 29 Zhu Xi–related cultural heritages. It provides a geographic-based online experience service for the cultural relics related to Zhu Xi, which integrates tourist routes and guided tours. The distance control enables a natural transition between the oblique photographic model (describing the environmental information of the cultural heritage surroundings) and the reconstruction model (portraying the detailed information of the cultural heritage). It can also be combined with 3D glasses for on-line immersive visualization. This module also provides a map of Zhu Xi’s major lifetime footprints. We can click on the key node for quick access to the relevant cultural heritage scene.
5. Panoramic picture/video: The reconstruction of the model inevitably results in the loss of information about the cultural heritage, which can be supplemented by a panoramic image/video of the veritable scene. This module provides the possibility of viewing panoramic pictures/videos of cultural heritage. By clicking on the hotspots of the panoramic scenes, you can view more detailed information. Videos of intangible cultural heritage recorded in panoramic video format can provide additional perspectives on the cultural heritage itself.

#### Zhu Xi metaverse system

**Fig. 5:**
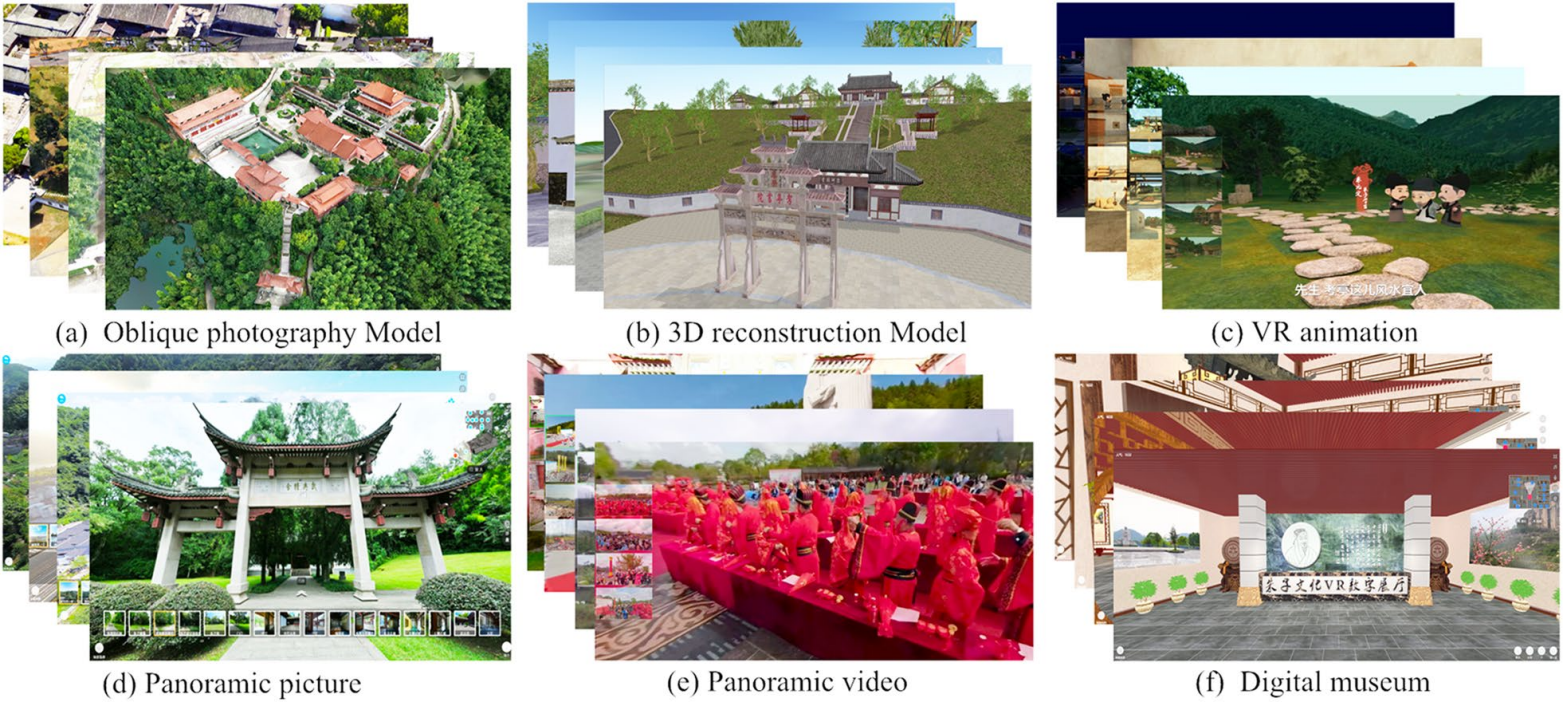
Digital products related to Zhu Xi cultural heritage

**Fig. 6:**
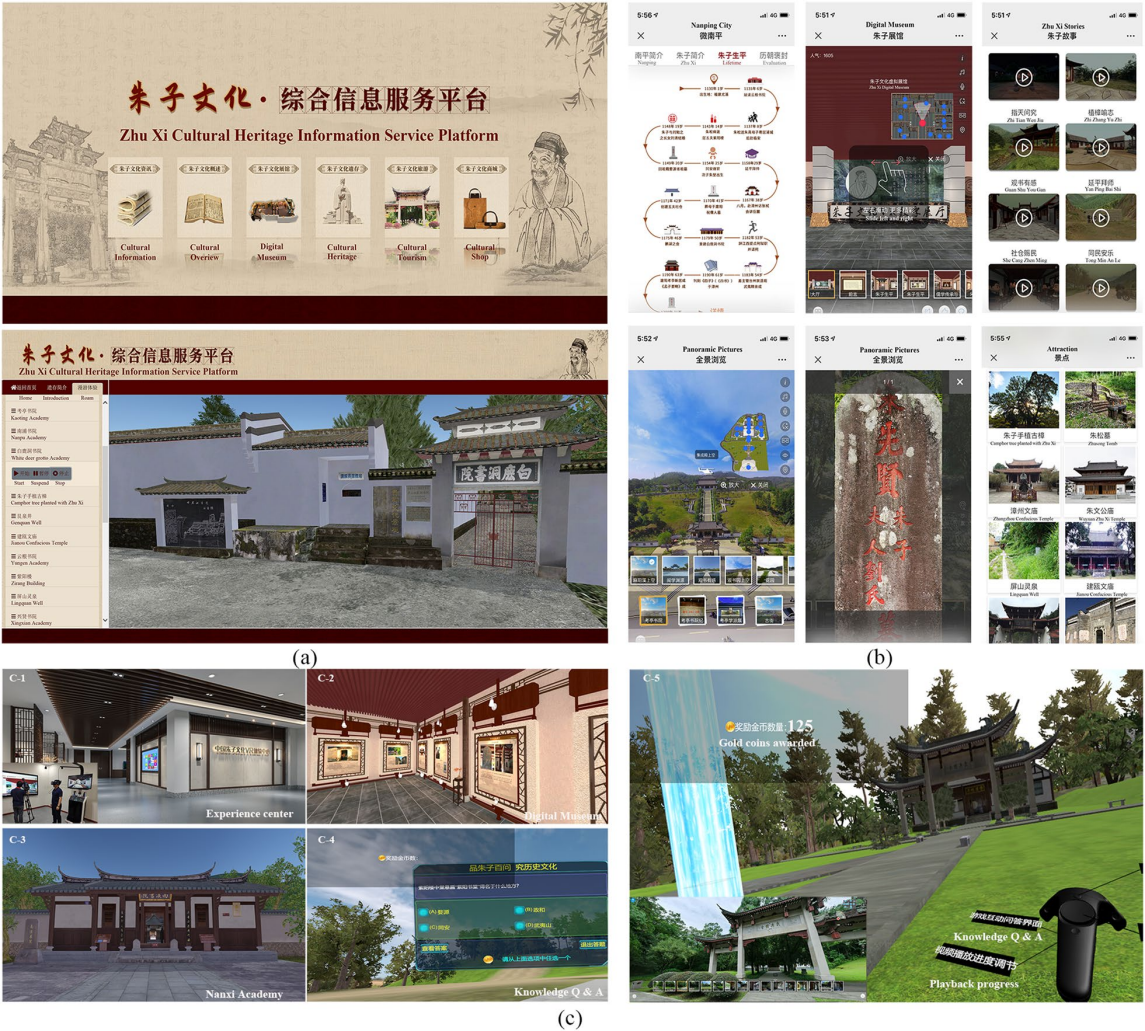
Zhu Xi cultural heritage information service platform: (a) web service; (b) mobile service; (c) metaverse system

The Zhu Xi metaverse system explores the co-existence of physical-virtual reality stages. There were five key VR components (digital museum, cultural heritage, anecdotes, four rites and cultural tourism) that resulted from the product creation used in this paper. The components are interoperable with each other. With the help of a VR helmet and control handle (Fig. 6(c-1)), the autonomous roaming service can enter the Zhu Xi digital museum (Fig. 6(c-2)), just like entering a real physical exhibition hall. Visitors can go anywhere they like. By touching the exhibit with the control handle, the exhibit can be enlarged or provide more detailed multimedia information (video, 3D model, etc.). Based on the collected 3D model data, we first constructed a large VR scene according to the general geographic environment, then placed, one-by-one, the Zhu Xi cultural heritage based on his life in the VR scene and fused it in the surrounding environment. Make the virtual environment as similar as possible to the real one. In the system, one can navigate from the Nanxi Academy (Zhu Xi was born, Fig. 6(c-3)) to the Cemetery of Zhu Xi (Zhu Xi was die). Follow the timeline for a complete tour of the cultural heritage sites left behind by Zhu Xi. At each site, we have set up trigger signs (e.g., Blue light pillar, Fig. 6(c-5)) for a more in-depth view of the physical appearance of the site (panoramic images or panoramic video), the story of the site happened (VR animation) and the related intangible cultural heritage (Four rites, festival, wedding ceremony, teacher worship ceremony, and capping ceremony). You can view this information by approaching the signs with the control handle and pulling the trigger button. After browsing, you can return to the cultural heritage virtual scene again by using the back button on the control handle. The system has also provided a competition project about relevant cultural knowledge (Fig. 6 (c-4)). The experiencer can answer questions about Zhu Xi in the specific scene location and receive credits and rewards. Based on the user system of VR experience system, the credits got in the system can be exchanged for tickets to Zhu Xi culture-related tourist attractions or Zhu Xi cultural creative products, etc.

#### Tourism service system

This paper devises a process-wide cultural tourism information service system. It caters to the needs of self-drive tourists and provides services at different stages of the tour.

1. Pre-tour: The paper employs a tourism information service subsystem to provide a more comprehensive understanding of cultural heritage related information prior to the tour. This provides cultural tourism information services for tourists, including attractions, itineraries, restaurants, accommodation, ticket prices, travelogues, panoramic pictures, panoramic videos, and so on. Besides this, the cultural experience system also allows visitors to learn more. We also employ a tour itinerary planning subsystem that allows for a rapid personalization of tour itineraries based on visitor preferences and supports optimization based on location and traffic information. Itineraries can be quickly customized by destination and keyword. Information on tickets, restaurants, and accommodation can also be added to the itinerary. You can make choices among attractions to quickly adjust the itinerary order. You can also add your favorite attractions, delete the ones you are not interested in, and organize your trip based on the suggested length of your visit and transport information.
2. Mid-tour: Based on pre-tour customized itineraries, we provide full navigation services during the tour, including itinerary navigation, automated voice guidance in scenic spot, support for real-time change of travel itineraries, traffic information queries and one-touch alarm services in case of emergency. During the trip, the subsystem provides recommendations for POI information around the route, including attractions, restaurants, accommodation and public facilities (e.g., toilets, car parks, petrol stations, banks, pharmacies, etc.).
3. Post-tour: After the tour, when you have added a huge number of beautiful photos to your phone and have nowhere to vent your excitement, you can share your travelogue via the Travelogue Sharing subsystem. The travelogue supports the input of texts or photos, which could be associated with the location information of the photos. You can create a personalized trail of all your travels and can forward the travelogues to other social media platforms with one click. We also offer an online e-shop for cultural products that you may not purchase on time for your trip. This provides a regional e-commerce platform for Zhu Xi–related cultural and creative products, regional characteristic cultural products, and local specialty products.

## Results and discussion

Based on the MDF-HF framework, we have completed the digitization of Zhu Xi’s cultural heritage resources. Part of the digitalized products are shown in Fig. 5. The digitized result is a thorough sorting and integration of Zhu Xi's cultural resources. It also provides multimodal contents such as oblique photographic models, 3D reconstruction models, panoramic pictures, panoramic videos and original 3D point cloud information, which provide accurate information for all-round documentation and research of cultural heritage. Based on the digitized documents, we further developed the ZXCHIS. The results are shown in Fig. 6. The public can access this via the web (http://zzwh.whlyw.net) or use their smartphone (http://zzwh.whlyw.net/mobile). You can also experience cultural resources in a more immersive way with the help of somatic interactive devices at the metaverse or VR experience center. To evaluate the ZXCHIS, we conducted a questionnaire survey. The results of questionnaires used for user studies are presented in Table 3. The total number of valid returns was 126, we found most of the users agree the platform is helpful and significant. This also reflects the validity of the digitization framework used in the paper. The question with the highest user recognition is “the platform has an enhancing effect on the preservation and promotion of the cultural heritage of Zhu Xi culture”. In response to the question “virtual scenes incorporating oblique models and 3D models can give you a better understanding of cultural heritage” approval rating of only 90%, and 7% chose “not sure”. Follow up through further interviews, they voted this way for 2 main reasons: Some users believe that a single oblique model or 3D reconstruction model cannot provide a comprehensive understanding of the culture, and that more expertise is needed to complement it. This proves that digitization of cultural heritage needs to consider multimodal data. Some responses to this access interface are complicated to operate, resulting in not getting accurate perceptions.

Economic and tourism development inevitably results in damages to cultural heritage. The acquisition of high-fidelity, high-precision documentation of cultural heritage is an important task in cultural heritage conservation. If the cultural heritage is destroyed (e.g., Notre-Dame de Paris fire), these digital documents will be the most valuable material for restoring it. The conflict between development and preservation of cultural heritage has never been reconciled. How to make good use of the results of digital documentation to realize the needs of many parties such as cultural research, heritage conservation, tourism development, and cultural value-added. This is something to consider before digitizing cultural heritage.

Each digital technology has strengths and weaknesses, the incorporated multiple methods offer the optimal choice. The MDF-HF framework considers both multimodal data collection and the need for forward-looking presentation techniques. It is the valid framework that we have revised and finalized. It has proved effective for digitizing Zhu Xi’s cultural heritage. This framework is an attempt to the digitization of historical figures. Further optimization may be required for other types of cultural heritage. We have summarized some of the empirical parameters and considerations in the digitization process. Each parameter is also the best result of our iterative adjustments. But rigorous objective validation requires more work. The digital research and tourism integration of Zhu Xi’s cultural heritage is still in its infancy. Parts of the cultural heritage may have to be extensively restored soon. This digital record will be the original digital documentation of this cultural heritage.

To ensure professionalism while making it interesting and educational, this paper offers two complementary solutions for different audiences. We have developed the Zhu Xi metaverse system to enable participants to get an immersive experience through interaction with the virtual environment or other operations. The research of the metaverse is also in a nascent stage. There is plenty of room for improvement in the systems we offer. Metaverse-oriented cultural heritage digitization flows still need to be further explored. The ultimate aim of the metaverse is to achieve the integration of virtual and real environments. At the experience level, we consider real content (panoramic images, panoramic videos, oblique photographic models, etc.) and reconstructed content (3D models, VR animation, etc.). This differs from traditional simulation games. Traditional simulation games focus on the entertainment function, while we focus on education. The game mainly uses 3D models and improves the visual experience through lighting rendering. But this is not enough for the cultural tourism experience. The scene needs to integrate more real-world experiences, and the interaction between the real-world and virtual experiences needs to be considered. The core feature of the metaverse system that distinguishes it from the game is the boundary between the virtual economy and the real economy. Specifically, people's economic activities in the game will not affect their real-world selves. While people's economic activities in the metaverse will affect their real-world selves. Users in the real-world enter the game and come out of the game, they are still themselves. When users in the real-world enter the metaverse and leave the metaverse, they are no longer themselves. Virtual human technology is considered an important part of the metaverse. For the VR animation, we created a 3D avatar of Zhu Xi. The relative construction of a more realistic Zhu Xi avatar is also the next step we need to consider for this system.

## Conclusions

Oriented towards historical figures, this paper designs a metaverse-based digital documentation framework for historical figures named MDF-HF. The framework ensures that the digitization of cultural heritage meets the needs of the construction of the metaverse. To test the validity of the framework, we have digitized almost all the major cultural heritage sites related to Zhu Xi. This work is groundbreaking and fills a gap in the digitization of cultural heritage in Zhu Xi cultural research. In terms of information services, we have designed different service options for different audiences. Following the metaverse-based concept, we have provided a metaverse version to create an immersive experience of Zhu Xi’s cultural heritage and promoted the research of the metaverse in the cultural heritage of historical figures.

## Data Availability

The datasets used and analyzed during the current study are available from the corresponding author on reasonable request.

## Abbreviations

3D: Three-dimensional
UVV: Unmanned aerial vehicle
XR: Extended reality
DOMs: Digital orthophoto maps
DSMs: Digital surface models
DTMs: Digital terrain models
VR: Virtual reality
AR: Augmented reality
MDF-HF: Metaverse-based digital documentation framework for historical figures
GIS: Geographic information system
IDM: Immersive digital museum
PVC: Polyvinyl chloride
POS: Position and orientation system
ZXCHIS: Zhu Xi cultural heritage information service platform
